# Elevated Plasma microRNA-206 Levels Predict Cognitive Decline and Progression to Dementia from Mild Cognitive Impairment

**DOI:** 10.3390/biom9110734

**Published:** 2019-11-13

**Authors:** Aidan Kenny, Hazel McArdle, Miguel Calero, Alberto Rabano, Stephen F. Madden, Kellie Adamson, Robert Forster, Elaine Spain, Jochen H. M. Prehn, David C. Henshall, Miguel Medina, Eva M. Jimenez-Mateos, Tobias Engel

**Affiliations:** 1 Department of Physiology and Medical Physics, Royal College of Surgeons in Ireland, Dublin D02 YN77, Ireland; aidankenny@rcsi.ie (A.K.); dhenshall@rcsi.ie (D.C.H.); 2 School of Chemical Sciences, Dublin City University, Dublin 9, Ireland; hazel.mcardle@dcu.ie (H.M.); Kellie.Adamson@dcu.ie (K.A.); elaine.spain@dcu.ie (E.S.); 3 Centro de Investigación Biomédica en Red de Enfermedades Neurodegenerativas (CIBERNED), 28031 Madrid, Spain; mcalero@isciii.es (M.C.); arabano@fundacioncien.es (A.R.); mmedina@ciberned.es (M.M.); 4 Carlos III Institute of Health, 28220 Madrid, Spain; 5 Centro de Investigación en Enfermedades Neurológicas (CIEN) Foundation, Queen Sofia Foundation Alzheimer Center, 28031 Madrid, Spain; 6 Data Science Centre, Royal College of Surgeons in Ireland, D02 YN77 Dublin, Ireland; stephenmadden@rcsi.ie; 7 School of Chemical Sciences, National Centre for Sensor Research, Dublin City University, Dublin 9, Ireland; robert.forster@dcu.ie; 8 FutureNeuro SFI Research Centre, Dublin D02 YN77, Ireland; 9 Discipline of Physiology, School of Medicine, Trinity College Dublin, The University of Dublin, Dublin D02 PN40, Ireland

**Keywords:** Alzheimer’s disease, prodromal, prognosis, microRNA, microfluidic device

## Abstract

The need for practical biomarkers for early diagnosis of Alzheimer’s disease (AD) remains largely unmet. Here we investigated the use of blood-based microRNAs as prognostic biomarkers for AD and their application in a novel electrochemical microfluidic device for microRNA detection. MicroRNA transcriptome was profiled in plasma from patients with mild cognitive impairment (MCI) and AD. MicroRNAs Let-7b and microRNA-206 were validated at elevated levels in MCI and AD, respectively. MicroRNA-206 displayed a strong correlation with cognitive decline and memory deficits. Longitudinal follow-ups over five years identified microRNA-206 increases preceding the onset of dementia. MicroRNA-206 was increased in unprocessed plasma of AD and MCI subjects, detected by our microfluidic device. While increased Let-7b levels in plasma may be used to identify patients with MCI, changes in plasma levels of microRNA-206 may be used to predict cognitive decline and progression towards dementia at an MCI stage. MicroRNA quantification via a microfluidic device could provide a practical cost-effective tool for the stratification of patients with MCI according to risk of developing AD.

## 1. Introduction

Alzheimer’s disease (AD) is the primary cause of dementia with over 46 million affected worldwide. The incidence of dementia is projected to continue growing to epidemic levels (> 131 million) by 2050 with an aging global population [1].

Despite this, developments in AD therapeutics have been fruitless with no therapy targeting the underlying disease pathogenesis having ever passed phase 3 clinical trials [2]. This has cast significant doubts over the current paradigms used in AD research and therapy development, specifically AD pathogenesis and the timing of treatment. The advanced stages of AD, at which clinical trials are applied, are often cited as a major contributor to the serial failures of AD therapeutic trials [3,4]. Recent studies indicate that the pathogenesis of AD precedes the onset of dementia and even minor cognitive deficits by 10 to 20 years [5,6]. The underlying processes which drive AD-associated neurodegeneration build slowly over time from a clinically silent stage progressing to a prodromal stage with mild cognitive impairment (MCI) and eventually dementia onset [5]. Much of the damage is thought to be irreversible upon the onset of dementia, thus limiting any potential impact of a therapy targeting the pathogenic processes [7]. Consequently, the focus has now shifted to implementing therapies at earlier stages of the disease offering a better opportunity for effective treatment and greater retention of cognitive function [8]. Identifying individuals at the prodromal stages of AD remains a major challenge with current diagnostic methods either lacking the sensitivity to accurately distinguish early stages of AD from age-related cognitive deficits or are impractical for a widespread application [9]. For these early stages of AD, neurological imaging of AD biomarkers [10,11] and cerebrospinal fluid (CSF) protein assays are among the few techniques implemented to detect prodromal AD [12]. However, the implementation of these methods is impractical for large populations due to the high cost of neuroimaging techniques [13], and the invasiveness of CSF collection [14]. Thus, the development of cost-effective biomarkers capable of predicting the conversion to dementia from a less invasive biofluid such as blood would be a significant aid to the development of AD therapeutics.

One class of molecules which have been increasingly implicated in AD and other neurodegenerative diseases are small non-coding microRNAs (miRNAs). MiRNAs act in the cell as post-transcriptional regulators of protein expression and, due to their multi-targeting nature, are capable of targeting entire molecular pathways. MiRNAs are most abundant in the CNS and are critical to neuronal development and functions including neurogenesis, dendritic development and synaptic plasticity [15]. Distinct miRNA transcriptomes have been well documented in diseased brains including AD with changes also being detectable in peripheral biofluids [16,17,18,19]. MiRNAs are an appealing source of biomarkers due to their stability in extracellular environments [20], reactive nature [19] and ease of quantification (e.g., RT-qPCR). In the present study, we identified miRNA biomarkers in plasma capable of differentiating between aged controls and patients with MCI and AD and evaluated the prognostic potential of miRNAs to predict cognitive decline using samples from a five-year longitudinal follow-up study [21].

## 2. Materials and Methods

### 2.1. Participants

All subjects provided their written informed consent for inclusion before they participated in the study. The study was conducted in accordance with the Declaration of Helsinki, and the protocol was approved by the Ethics Committee of the Carlos III Institute of Health (CEI PI 46_2011-v2015). Participants were grouped into a discovery cohort and a longitudinal cohort (Table 1). The discovery cohort included 31 control subjects and 30 patients with MCI, recruited from the Vallecas project (Madrid, Spain), a 5-year single-centre longitudinal community-based study, with yearly neurological and neuropsychological assessments, as well as genetic testing at the first (baseline) visit. Inclusion/exclusion criteria have been described previously [21]. Collections of biological samples and clinical data were all performed at CIEN Foundation (Madrid, Spain). Retrospective samples from AD subjects (*n* = 25) were obtained from the onsite BT-CIEN biobank located at the same Centre [22]. Exclusion criteria included lack of AD pathology at post-mortem histology or primary pathology resultant from non-AD dementia (e.g., Vascular dementia, Lewy-body dementia, Lateral sclerosis). The longitudinal cohort included 18 subjects within 3 groups with distinct cognitive progressions from cognitively healthy or MCI to dementia or stable MCI with plasma samples collected at 1–2-year intervals over a 5-year time period.

### 2.2. Sample Collection

Blood samples were collected with citrate and centrifuged at 2280 *g* for 10 min followed by 10 min at 13,000 rpm within 1 h of collection. Platelet-free plasma was then stored at −80 °C. All blood samples (Control, MCI, and AD) were collected at the same Center (CIEN Foundation, Madrid, Spain), by the same group of technicians and using the same protocol [21], thus assuring that sample processing is comparable. All samples are stored under the same conditions following identical protocols. Timeframes of storage from controls have been selected to match the timeframes of AD samples.

### 2.3. Neuropsychological Assessment

A neuropsychological test battery was applied by neuropsychologists, evaluating visual perception, attention, memory, language, praxis and executive function including tests of cognitive performance: Mini Mental State Exam (MMSE), Free and Cued Selective Reminding Test (FCSRT), and functional scales such as Clinical Dementia Rating (CDR) and Functional Activities Questionnaire (FAQ) [23]. MCI patients were grouped according to cognitive decline based on MMSE change over time (ΔMMSE): subjects with a decreased MMSE score of ≥ 3 points over 4 years with a final MMSE score ≤ 24 were grouped as “MCI decliners” and subjects with MMSE reduction < 3 and/or MMSE > 24 were grouped as “MCI stable”. MCI subjects were also grouped based on age-adjusted free cued selective reminding test (FCSRT)–free recall [24] for high-risk MCI to dementia progression. Eleven out of 30 MCI subjects were omitted from analysis due to drop-out before year 4 in addition to 2 subjects which were removed as outliers [25,26].

### 2.4. RNA Extraction and OpenArray Profiling

Small RNAs were isolated from 50 µL of plasma using Serum/Plasma small RNA kit (Qiagen, West Sussex, UK) to produce small RNA suspended in RNase free water. MiRNA profiling was performed using the OpenArray platform (ThermoFisher Scientific, Waltham, MA, USA) [27,28]. OpenArray reverse transcription reaction was performed according to the manufacturer’s protocol using small RNA pooled from 10 samples. cDNA was pre-amplified and loaded onto the OpenArray automatically by the OpenArray AccuFill System (ThermoFisher Scientific) and run on a QuantStudio 12K Flex Real-Time PCR system. A total of 754 human miRNAs were amplified from each sample together with 16 replicates of 4 internal controls (ath-miR159a (negative control), RNU48, RNU44, and U6 rRNA). OpenArray Ct values were normalised to the global mean (GMN) [27].

### 2.5. Individual RT-qPCR

OpenArray results were validated using small-scale RT-qPCR [29]. Extracted small RNA was reverse transcribed using Reverse Transcription kit (ThermoFisher Scientific) and Taqman primers (miR-206, ID: 000510, Let-7b, ID: 002619, miR-135a, ID: 000460) and quantified by RT-qPCR. MiR-378 (ID: 002243) was selected as endogenous control showing stable expression between conditions (Relative expression: Control (1 ± 0), MCI (1.2 ± 0.58), AD (1.1 ± 0.25)). Ct values were normalised to miR-378 using the 2^−ΔΔCt^, where ΔΔCt = ΔCt miRNA sample X – ΔCt miRNA reference sample and ΔCt = Ct miRNA (miR-206, Let-7b, or miR-135a) - Ct miR-378. Cut-off for detectable Ct values were set at 35 Ct. MiR-206 concentration in plasma was calculated via miR-206 standard curve generated by using known concentrations of miR-206 mimics (miRVana mimic hsa-miR-206 (MC10409), ThermoFisher Scientific) quantified simultaneously with patient samples by RT-qPCR. Ct values of serial dilutions were plotted against fM concentrations on a logarithmic scale and sample concentrations extrapolated.

### 2.6. MiRNA Electrochemical Detection

Platinum nanoparticles (PtNPs) (50–70 nm) were purchased from Strem Chemicals. The oligonucleotides (purity > 98%) were purchased from Eurogentec. The PtNPs were uniformly functionalised with the probe strand miRNA to achieve a closely packed nucleic acid layer (density approximately 1 × 10^10^ probes/cm^2^), by incubating the PtNPs with a 1 µM solution of the probe miRNA strand at 37 °C overnight. A three-electrode electrochemical cell was used at a temperature of 22 ± 2 °C. The working electrode was a freshly polished 2 mm diameter planar gold disc electrode (GDE). The counter electrode was a large area coiled platinum wire and a silver/silver chloride (Ag/AgCl in 3 M KCl) acted as reference. All amperometric detection measurements were performed using a CH Instruments, Model 760D, electrochemical workstation. A sandwich assay was formed on the surface of a freshly polished GDE. A monolayer of capture strand miRNA (which is complementary to part of the target, miR-206) was formed on the GDE by immersing in a 1 µM solution of the capture oligo dissolved in Denhardt’s buffer at 37 °C for 5 h. Hybridisation of the target miRNA to the immobilised capture strand was performed by incubating the electrode in the raw plasma samples at 37 °C for 3 h. The PtNP-labelled probe miRNA was hybridised to the remaining complementary section of the target miRNA for 5 h at 37 °C. Between each step, the electrode was thoroughly washed with RNase free water. Following the formation of the sandwich assay, the modified electrode was placed in 0.1 M H_2_SO_4_ and a potential of −0.25 V was applied. The resulting current was measured. After 10 min H_2_O_2_ was added to give a final concentration of 200 µM, the current associated with the reduction of H_2_O_2_ was measured after 30 min. The analytical response is the difference in current before and after H_2_O_2_ addition [30].

### 2.7. Statistical Analysis

*T*-test, Mann–Whitney, and Kruskal-Wallis were used to assess differences in demographic, clinical phenotypes, genotype, and miRNA expression between groups. *p* < 0.05 was considered statistically significant and adjusted for multiple comparisons using Bonferroni correction. Correlations between variables were assessed using Pearson’s method. All groups were evaluated by Grubb’s test and identified outliers were omitted from analysis. Receiver operating characteristic curve (ROC) analysis was used to determine capability of miR-206 in differentiating between MCI subjects with FCSRT-F below cut-off vs. MCI subjects with FCSRT-F scores above cut-off and MCI subjects grouped as “MCI decliners” vs. “MCI stable”. Graphpad Prism version 5.01 was used for statistical analysis.

## 3. Results

### 3.1. MicroRNA Profiling in Patient Plasma

**Figure 1:**
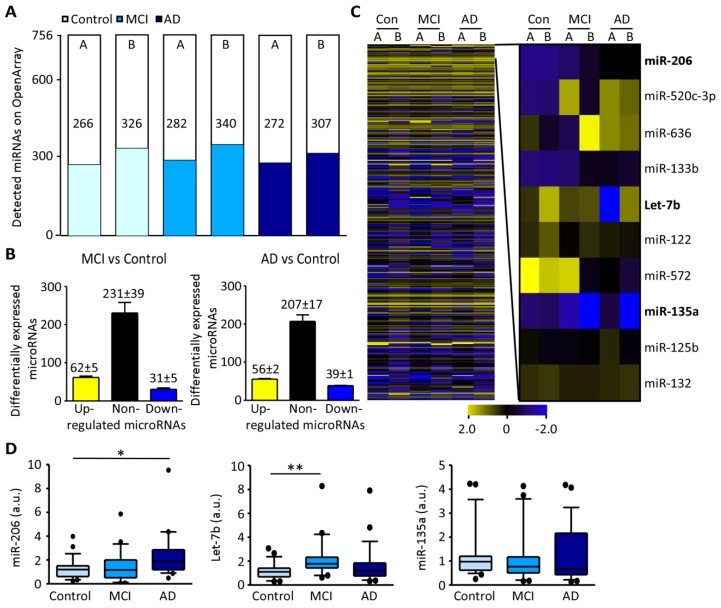
Relative miRNA expression levels in plasma from patients with MCI and AD compared to control: (**A**), Number of miRNAs detected by OpenArray from two independent experiments (A and B) (control (light blue), MCI (blue) and AD (dark blue)), (*n* = 10 per pool). (**B**), Graph showing the number of up-regulated miRNAs (yellow), non-regulated miRNAs (black) and down-regulated miRNAs (dark blue) in MCI (left) and AD compared to control (right). (**C**), Heat map of miRNA expression according to each condition (control, MCI, AD) of each individual array (left) and amplification of specific miRNAs including miR-206, Let-7b, and miR-135a (right). (**D**), Individual RT-qPCR in validation cohort shows increased miR-206 plasma levels in AD (left, MCI vs. control *p* = 0.945, AD vs. control *p* = 0.0168, Mann-Whitney, *n* = 29 (control), 25 (MCI), 23 (AD)) and increased Let-7b plasma levels in MCI (middle, MCI vs. control *p* = 0.0026, AD vs. control *p* = 0.429, *n* = 23 (control), 20 (MCI), 23 (AD)). No differences were observed for miR-135a between conditions (right, MCI vs. control *p* = 0.267, AD vs. control *p* = 0.276, *n* = 27 (control), 25 (MCI), 21 (AD)). In the scatter-box, the horizontal line represents the median and the top and bottom boxes represent the 75^th^ and 25^th^ percentiles, respectively. The whiskers above and below extend the most extreme point no longer than 1.5 times the interquartile range from the box. Abbreviations: MCI: Mild cognitive impairment, AD: Alzheimer’s disease. * *p* < 0.025, ** *p* < 0.005.

Profiling of miRNAs in plasma from subjects pooled according to disease state was carried out using the OpenArray platform [29] on two independent arrays (Array A and Array B, control (*n* = 10), MCI (*n* = 10) and AD (*n* = 10) per array). This identified 266, 282, and 272 miRNAs detected in control, MCI and AD respectively in Array A and 326, 340, and 307 miRNAs in control, MCI and AD respectively in Array B (Figure 1A). For screening of biomarker candidates, miRNAs were grouped as “increased” with relative expression (RE) > 2.0 or “decreased” with RE < 0.5 compared to control. The majority of miRNAs remained within 2–0.5 RE in both arrays with an average of 62 ± 5 increased and 31 ± 5 decreased in MCI and 56 ± 2 increased and 39 ± 1 decreased in AD (Figure 1B). Identified miRNA biomarker candidates from the profile included: miR-206, miRNA upregulated in MCI and AD in both arrays, Let-7b, increased in MCI, and miR-135a, miRNA downregulated or undetectable in MCI and AD while present at high levels in control in both arrays (Figure 1C and Supplementary Material Figure S1A). Validation by individual RT-qPCR confirmed significant increases in miR-206 plasma concentration in AD relative to controls (*p* = 0.0168) but no significant increase in MCI (*p* = 0.945). Let-7b was increased in MCI (*p* = 0.0026) with no statistical difference in AD (*p* = 0.429). MiR-135a showed no significant changes from control in MCI (*p* = 0.267) or AD (*p* = 0.276) (Figure 1D and Supplementary Material Figure S1B). The increase of total miR-206 concentration in patient plasma was confirmed in selected control, MCI and AD samples (*n* = 5 per group) from the discovery cohort extrapolating against standard curve of miR-206 oligonucleotides. MiR-206 was increased in MCI (*p* = 0.0317) and in AD (*p* = 0.0079) compared to control (Supplementary Material Figure S1C,D).

In summary, our results show increased Let7b levels in the plasma of patients with MCI and increased miR-206 levels in the plasma of patients with AD.

### 3.2. Increased miR-206 Plasma Levels Predict Cognitive Decline during MCI

Next, our selected miRNAs were evaluated for relationships with cognitive decline and conversion from MCI to dementia, based on a 4-year longitudinal evaluation. MCI subjects were grouped according to FCSRT Free score cut-offs for increased risk of AD and cognitive decline based on stable or deteriorating MMSE score over 4 years (Table 2). MiR-206 was significantly higher in MCI patients grouped by FCSRT for high risk of dementia (*p* = 0.0174) and in MCI patients with deteriorating MMSE scores over the 4 years (*p* = 0.0055) (Table 2). Let-7b and miR-135a displayed no significant differences in FCSRT or MMSE groups (Table 2).

**Figure 2:**
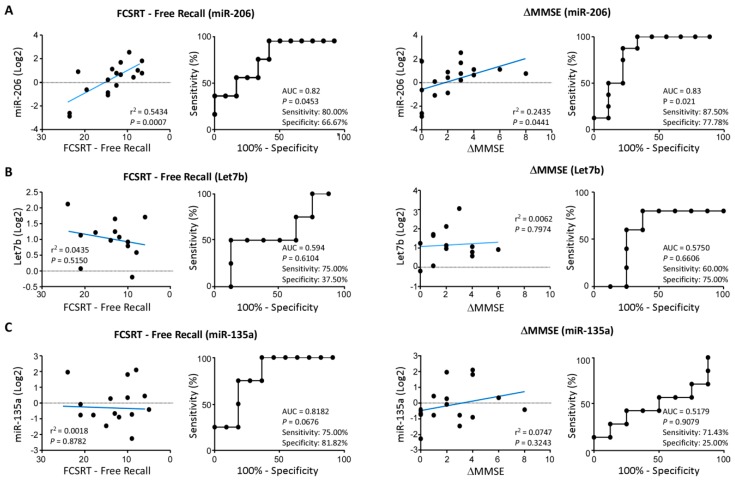
MiR-206 correlation with cognitive decline using the Free and Cued Selective Reminding Test (FCSRT) and Mini-Mental State Examination (MMSE): (**A**), MiR-206 relative expression is strongly associated with FCSRT-free recall score (*p* = 0.035, *n* = 17) and decreases in MMSE score (*p* = 0.0004, *n* = 17) over 4 years. ROC analysis shows miR-206 had 80.00% sensitivity and 66.67% specificity for identifying high risk dementia subjects scoring below age adjusted FCSRT-free recall cut-off and 87.50% sensitivity and 77.78% specificity to predict cognitive decline using the MMSE test. (**B**), Relative expression of Let-7b when compared to cognitively healthy controls showed no correlation with FCSRT-free recall (*p* = 0.515, *n* = 13) and subjects grouped by age adjusted FCSRT-free recall cut-offs showed no significant difference in Let-7b between groups (*p* = 0.6104, *n* = 9 (above cut-off), 4 (below cut-off)). Relative expression of Let-7b compared to cognitively healthy controls showed no correlation with ΔMMSE in MCI subjects (*p* = 0.7974, *n* = 13) and subjects grouped by changes in MMSE over 4 years showed no significant difference in relative expression of Let-7b between groups (*p* = 0.661, *n* = 8 (stable MCI), 5 (MCI decliners)). (**C**), Relative expression compared to cognitively healthy controls of miR-135a showed no correlation to FCSRT-free recall score in MCI subjects (*p* = 0.878, *n* = 15) and subjects grouped by age adjusted FCSRT-free recall cut-offs showed no significant difference in miR-135a between groups (*p* = 0.0676, Mann-Whitney, *n* = 11 (above cut-off), 4 (below cut-off)). Relative expression of miR-135a compared to cognitively healthy controls showed no correlation with ΔMMSE in MCI subjects (*p* = 0.324, *n* = 15) and subjects grouped by changes in MMSE over 4 years showed no significant difference in relative expression of miR-135a between groups (*p* = 0.908, *n* = 8 (MCI stable), 7 (MCI decliners)).

When miR-206 plasma levels were correlated to FCSRT-Free recall score, a strong correlation was observed between memory deficits and increases in miR-206 (*p* = 0.0007; r^2^ = 0.5434) (Figure 2A). MiR-206 increases also showed a strong correlation with cognitive decline measured via MMSE scores (*p* = 0.044, r^2^ = 0.2435) (Figure 2A). Let-7b and miR-135a displayed no correlation with FCSRT or MMSE scores (Figure 2B,C).

ROC analysis showed a good accuracy of miR-206 in plasma for identifying FCSRT deficits at MCI (*p =* 0.0277, AUC = 0.82) and for identifying MCI subjects with declining MMSE scores over 4 years (*p* = 0.021, AUC = 0.83) (Figure 2A). Notably, the inclusion of early drop-outs with “last observation carried forward” (subject is included in analysis with last analysis before dropout taken as the endpoint) increased correlations and sensitivity and specificity of miR-206 for MCI with cognitive decline profoundly (*p* = 0.001, AUC = 0.90) (Supplementary Material Figure S2). In contrast to miR-206, ROC analysis carried out for Let7b and miR-135a showed no distinction between MCI and control patient groups (Figure 2B,C).

**Figure 3:**
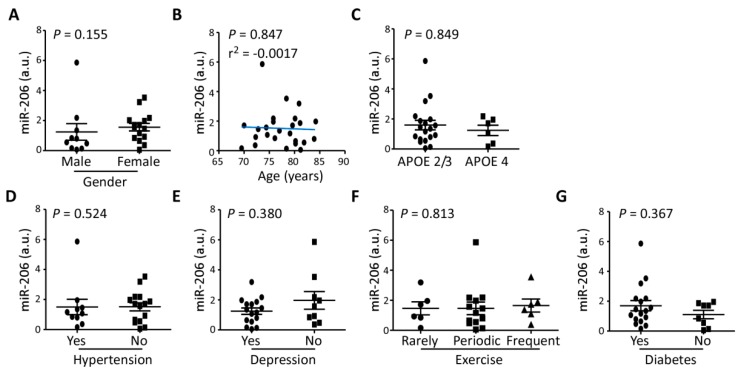
Clinical covariates and relative expression of miR-206: Relative expression of miR-206 in MCI showed no significant difference due to (**A**), gender (*p* = 0.155, Mann-Whitney, *n* = 10 (male), 15 (female)), (**B**), age (*p* = 0.847, *n* = 25), (**C**), APOE genotype (*p* = 0.849, Mann-Whitney, *n* = 19 (APOE 2/3), 6 (APOE 4)), (**D**), hypertension (*p* = 0.524, Mann-Whitney, *n* = 9 (Yes), 16 (No)), (**E**), depression (*p* = 0.380, Mann-Whitney, *n* = 16 (Yes), 9 (No)), (**F**), level of physical activity (*p* = 0.813, Kruskal-Wallis, *n* = 6 (Rarely), 13 (Periodic), 6 (Frequent/Always)), and (**G**), diabetes (*p* = 0.367, Mann-Whitney, *n* = 17 (Yes), 8 (No)).

No correlation of miR-206 with potential confounding factors was identified within the MCI group including gender (*p* = 0.155) (Figure 3A), age (*p* = 0.847, r^2^ = 0.041) (Figure 3B), APOE genotype (*p* = 0.849) (Figure 3C), hypertension (*p* = 0.524) (Figure 3D), depression (*p* = 0.380) (Figure 3E), exercise (*p* = 0.813) (Figure 3 F), and diabetes (*p* = 0.367) (Figure 3G).

Thus, miR-206 was the only candidate miRNA correlating with two independent cognitive assessments (FCSRT and MMSE), while also increased in plasma of patients with AD.

### 3.3. MiR-206 Plasma Concentration in Longitudinal Sampling

**Figure 4:**
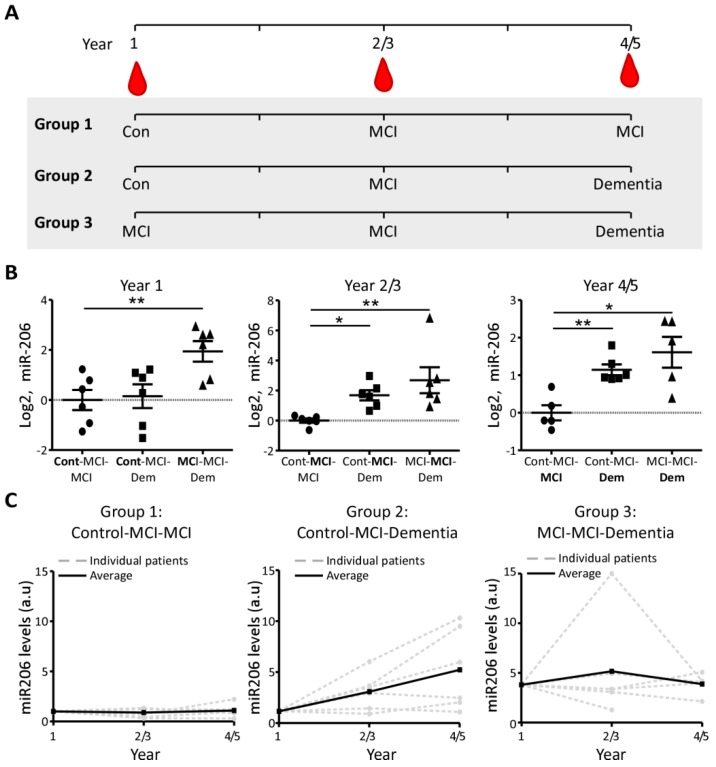
MiR-206 plasma concentration changes predict cognitive decline and development of dementia at mild cognitive impairment (MCI) stage: (**A**), Blood sampling was carried out annually from year 1 to year 4/5. Volunteers were divided into three groups based on MMSE and FCSRT results (Table 2). Group 1 includes control subjects who develop MCI during the 5 years. Group 2 includes subjects who have the normal cognitive ability in the first year but show cognitive deterioration in the follow-up visits to MCI and dementia. Group 3 includes MCI patients who developed dementia in year 5. (**B**), No differences were observed in miR-206 in year 1 between Group 1 and Group 2 (*p* = 0.801, *t*-test, *n* = 6). Patients from Group 3 showed higher miR-206 plasma levels when compared to stable control Group 1 (*p* = 0.0069, *t*-test, *n* = 6). Increased miR-206 plasma levels in patients at MCI stage (year 2/3) who developed dementia in later life when compared to Group 1 (Group 1 vs. Group 2, *p* = 0.0119 and Group 1 vs. Group 3, *p* = 0.0022, *t*-test, *n* = 6). Elevated miR-206 levels in plasma of subjects who developed dementia later in life in year 4/5 (Group 1 vs. Group 2, *p* = 0.0011, *t*-test, *n* = 6) and Group 1 vs. Group 3, *p* = 0.0159, *t*-test, *n* = 6). * *p* < 0.05, ** *p* < 0.01. (**C**), MiR-206 plasma levels in Group 1 subjects with stable MCI changed very little over the duration of the study. Average miR-206 plasma levels increased almost linearly in Group 2 subjects who progressed from control to dementia during the period of the study. MiR-206 was observed at an already elevated level in Group 3 without further increases in subjects progressing from MCI to dementia.

To validate the relationship of miR-206 levels to cognitive state and predicting changes in cognitive status, the plasma concentration of miR-206 was analysed in a longitudinal cohort of patients with repeated blood sampling from the same patient over time. Patients were split into three groups with distinct cognitive state progressions and miR-206 levels were analysed at three time-points. The three cohorts included “MCI stable”: Group 1 (control (year 1), MCI (year 3) and MCI (year 5)), “control converters”: Group 2 (control (year 1), MCI (year 3) and dementia (year 5)) and “MCI converters”: Group 3 (MCI (year 1), MCI (year 3), dementia (year 5)) (Figure 4A).

Comparison between cohorts at year one showed no differences in miR-206 levels between subjects at a healthy control state (Group 1 and 2) (*p* = 0.810), while patients already at MCI with altered progression towards dementia (Group 3) displayed significantly higher levels of miR-206 compared to both groups (*p* = 0.0069). At year 2/3, when all cohorts had entered MCI state, miR-206 was found to be elevated within both Group 2 (*p* = 0.0119) and Group 3 (*p* = 0.0022) compared to Group 1. At year 4/5, significant differences remained between groups where patients converted to dementia when compared to patients with stable MCI (Group 1 vs. Group 2, (*p* = 0.0011) and Group 1 vs. Group 3, (*p* = 0.0159)) (Figure 4B). Confirming these results, when analysing each individual subject longitudinally, stable MCI subjects in Group 1 displayed little to no change in expression of miR-206 over the 4/5 years, while in Group 2, 67% of subjects displayed miR-206 increases from year 1 to year 2/3 and 83% displayed increases at 4/5-year time-point. Patients in Group 3 increased miR-206 compared to all other cohorts at year 1 but showing no further increase in miR-206 over the 4/5 years (Figure 4C).

### 3.4. Prototype Clinical Assay for AD Risk

**Figure 5:**
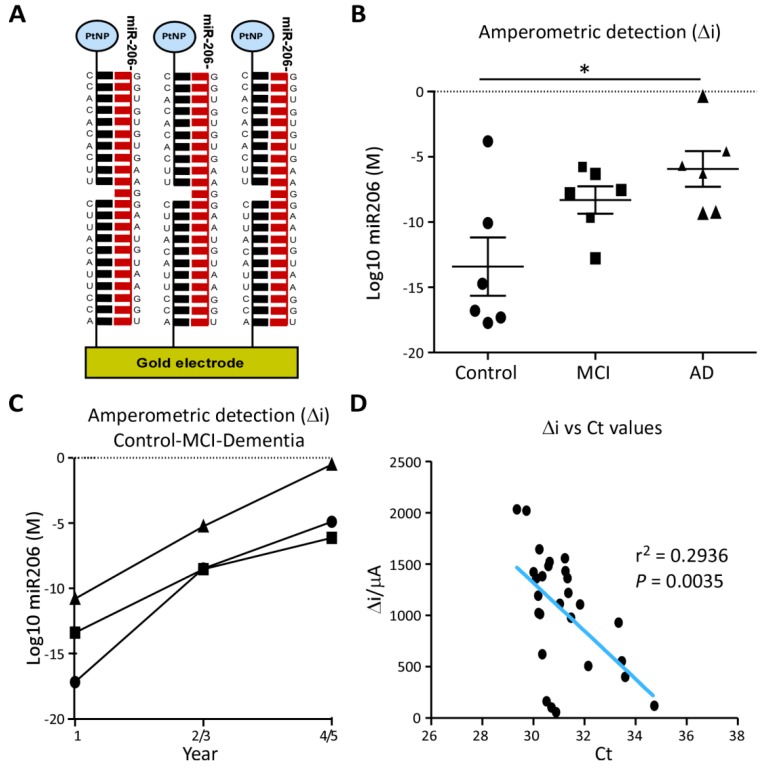
MiR-206 quantification by electrochemical microfluidic device: **(A**), Illustration of the electrochemical sandwich assay for the quantification of miR-206, composed of capture oligonucleotides affixed to a gold electrode and platinum nanoparticle (PtNP) labelled probe oligonucleotides complementary to miR-206′s sequence. (**B**), Molar concentration of miR-206 was measured by amperometric detection (Δi) with a prototype electrochemical sandwich assay in unprocessed plasma from Control, MCI and AD subjects (*n* = 6 per group) and 3 subjects at 2-year intervals from Group 2 of the longitudinal cohort. Concentration of miR-206 is increased in AD plasma compared to control (*p* = 0.0455, Mann-Whitney, *n* = 6) but not significantly increased in MCI (*p* = 0.0935, Mann-Whitney, *n* = 6). * *p* < 0.05. (**C**), Measurement of miR-206 in the longitudinal cohort showed an almost linear increase in miR-206 concentration over 5 years in all subjects. (**D**), Δi for miR-206 detected by electrochemical assay in unprocessed plasma plotted against Ct values from miR-206 RT-qPCRs performed on small RNA extraction from plasma from the same patient. Δi concentrations and Ct values showed a strong inverse correlation (*p* = 0.0035, r^2^ = 0.2936, *n* = 36).

MiR-206 was also evaluated as a biomarker using an oligonucleotide sandwich assay electrochemical detection system capable of detecting the ultralow concentration of miRNAs in raw plasma (Figure 5A and Supplementary Material Figure S3) [30]. By using raw plasma, this detection system requires less sample preparation, thereby reducing possible confounding factors when implementing miRNA-based diagnostic tests across different sites. Mirroring RT-qPCR results, miR-206 concentrations detected by the assay were elevated in AD samples compared to control (*p* = 0.0455) and, although not statistically significant, miR-206 levels in MCI were present at noticeably higher levels than control (*p* = 0.0935) (Figure 5B). The concentration of miR-206 in raw plasma was also increased over time in longitudinal case-series of individuals progressing from control to dementia (Figure 5C). Amperometric values from each subject showed a strong correlation with the RT-qPCR Ct values obtained from the same samples cross-validating the elevated levels of miR-206 plasma and the novel detection system (Figure 5D).

Taken together, our results suggest that the microfluidic device measuring miR-206 in plasma could be implemented in combination with other diagnostic tools for more efficient identification of prodromal AD for both improved clinical care and therapeutic trials, making the targeting of these preliminary stages of AD more feasible.

## 4. Discussion

In the present study, we investigated the potential of miRNAs as biomarkers for prodromal and pre-symptomatic stages of AD and tested novel methods for their detection which could be easily and cost-efficiently implemented on large populations. We identified increases in miR-206 during AD and in MCI subjects progressing towards an AD-like phenotype and increased Let-7b levels during MCI. These findings suggest miR-206 in blood is a viable biomarker for AD and predicting the conversion from MCI to dementia.

MiRNAs have been investigated as potential biomarkers for AD previously, with studies in blood and CSF [16,17,18,31,32,33]. MiR-135a, Let-7b, and miR-206 had all previously been investigated in CSF or blood-derived samples from AD patients. MiR-135a was identified as downregulated in probable AD subjects [31] mirroring our OpenArray results, however, not confirmed by individual RT-qPCR. Notably, Let-7b has been identified as downregulated in whole blood of AD patients previously [34], contrasting our plasma analysis where Let-7b showed no change in AD but was increased in MCI. This variation could be attributed to miRNA compositions in whole blood compared to plasma. Nevertheless, increased Let-7b at MCI stages suggests this miRNA to be a potential biomarker for the MCI state. Let-7b levels were, however, independent of eventual disease progressions from MCI.

MiR-206 has been previously identified as elevated in the serum of amnestic MCI (aMCI) subjects who progressed onto AD compared to stable aMCI subjects [35]. These findings parallel the observed miR-206 increases in cognitively declining MCI subjects in our study as well as the elevated miR-206 prior to dementia in our longitudinal cohort. These complementary findings in two entirely distinct populations (Madrid, Spain [21] and Heibei province, China [35]) strongly indicate that miR-206 changes are independent of ethnic and cultural factors, which have been shown to impact on biomarker profiles [36].

Analysis of miR-206 in the longitudinal cohort at 2-year intervals revealed a strong link between miR-206 and the cognitive state with miR-206 increases correlating strongly with worsening disease progression. MiR-206 was elevated in all MCI groups at least 2 years prior to dementia onset but no increases in miR-206 were detectable at the cognitively healthy state. Neurodegenerative mechanisms would almost certainly be present at this pre-clinical stage indicating miR-206 increases either occur later during neurodegeneration and/or are dependent on the breakdown of the blood-brain-barrier during MCI [37,38] enabling the increased miR-206 present in the central nervous system (CNS) to leech into peripheral circulation. MiR-206 increases being derived from the CNS is further supported by miR-206′s elevated presence in the cortex [39] and olfactory mucosa of AD patients [40]. One possible limitation of the study was the departure of subjects who developed dementia during the study from the Vallecas project, making probable AD diagnosis not feasible. To accommodate this, AD subjects with confirmed post mortem AD were included from the CIEN Biobank to identify miRNA biomarkers shared between AD and MCI [41]. Even though in the present study miR-206 has not been quantified in non-AD dementias, miR-206 has been identified as downregulated in Frontotemporal dementia patient plasma indicating miR-206 expression might be distinct in other dementias [42]. In addition, only a limited number of subjects in the discovery cohort progressed to dementia from MCI so far over the 4 years (*n* = 3), representing a potential limitation for a definite diagnosis of dementia and AD. In the absence of this, neuropsychological indicators collected over the duration of the study were used in each case-series to assess the likelihood of progression to dementia and identify potential pre-symptomatic AD. ΔMMSE over the 4 years was employed to evaluate the cognitive decline symptomatic of prodromal AD [43,44,45]. Our results indicate that higher levels of miR-206 were predictive for cognitive decline and onset of dementia in MCI subjects, miR-206 levels correlated strongly to declining MMSE scores over time. Age-adjusted FCSRT was employed as an evaluation of episodic and working memory (earliest areas of cognition damaged by AD [46]) and an indicator for likelihood of dementia onset [25]. MiR-206 was also elevated in MCI subjects below the FCSRT cut-off indicating that subjects with increased miR-206 were at high risk of developing dementia.

MiR-206′s presence and function in the CNS has only recently begun to be unravelled due to its transient expression in healthy brains, only being found at notable concentrations in injured brains [39]. The mechanisms behind this increase of miR-206 and its function in the CNS remain unclear but miR-206 has some notable putative targets which could greatly impact the CNS including Brain-derived neurotrophic factor (BDNF), Histone deacetylase 4 (HDAC4) and JunD [39,47,48]. In particular, miR-206′s targeting of BDNF would have a significant neurodegenerative effect due to its essential functions in the homeostasis of the CNS, being involved in neuronal survival, synaptic plasticity, dendritic branching, and regulation of neurotransmission [49,50]. Notably, decreases in BDNF protein and mRNA levels have been well documented in the brains of individuals with MCI and AD [51,52] with these decreases thought to be central to the synaptic loss and cell death which drives AD pathology. MiR-206′s silencing of BDNF has been identified in AD mouse models overexpressing mutated human amyloid precursor protein and has been shown to result in decreased spine density. On the other hand, inhibition of miR-206 via antagonist oligonucleotides resulted in improved memory function and increased neurogenesis via increased levels of BDNF suggesting miR-206 as a potential therapeutic target for AD [39].

Although current methods to analyse miRNAs have significant advantages in stability and ease of use, they are not a point-of-care device. To further the potential of miRNA as diagnostic biomarkers for AD, we have investigated the implementation of miRNA-based diagnostic with the above described electrochemical assay with unprocessed plasma. With this technique, we have established a simple rapid point-of-care assay requiring almost no sample preparation which could be implemented in the diagnosis of AD and the prediction of cognitive decline.

In conclusion, the present study demonstrates the diagnostic and prognostic potential of blood-based miRNAs for MCI and AD. The identification of miR-206 as a disease-stage specific biomarker and predictor for cognitive decline and memory loss may allow the development of a simple, minimally invasive and cost-effective test for AD development and prodromal dementia.

## Figures and Tables

**Table 1:** Demographic and clinical characteristics of patient cohorts.

**Discovery Cohort**	**Control (*n* = 31, CDR = 0)**	**MCI (*n* = 30, CDR = 0.5)**	**AD (*n* = 25, CDR ≥1)**	***p* Value**
Age, mean (SD) [range]	75.0 (4.7) [69–86]	76.8 (4.0) [69–84]	84.6 (3.5) [77–90]	<0.0001 ***
Male sex, No. (%)	17 (54.8)	13 (43.3)	4 (16)	0.02 *
Educational level mean (SD)	11.8 (5.6)	7.0 (4.3)	3.8 (1.1)	<0.0001 ***
1 or 2 APOE4 alleles	4.0	9.0	10.0	0.05
Diabetes Mellitus, %	12.9	26.7	15.8	0.37
Hypertension, %	48.4	53.3	57.9	0.86
Physical Exercise frequency, mean (SD) [range]	0.6 (0.4) [0–1]	0.4 (0.4) [0–1]	NA	0.0568
Never/Rarely (0)	7.0	15.0	NA	
Sometimes (0.5)	10.0	9.0	NA	
Frequently/Always (1)	14.0	6.0	NA	
Annual visits completed, (SD) [range]	3.8 (1.1) [2–5]	3.8 (1.4) [2–5]	NA	0.83
MMSE mean (SD)	28.7 (1.5)	26.6 (2)	NA	<0.0001 ***
**Longitudinal Cohort**	**Group 1: Control-MCI-MCI (*n* = 6)**	**Group 2: Control-MCI-Dementia (*n* = 6)**	**Group 3: MCI-MCI-Dementia (*n* = 6)**	***p* Value**
Age, mean (SD) [range]	74.0 (3.2) [69.6–76.9]	77.3 (3.8) [70.7–81.8]	75.8 (3.6) [72.3-82.3]	0.338
Male sex, No. (%)	2 (33.3)	1 (16.6)	4 (66.6)	0.213
Hypertension, %	32.2	50	32.2	0.802
Physical Exercise frequency, mean (SD) [range]	0.5 (0.4) [0–1]	0.6 (0.5) [0–1]	0.4 (0.4) [0–1]	0.79
Never/Rarely (0)	2	2	2	
Sometimes (0.5)	2	1	3	
Frequently/Always (1)	2	3	1	
Annual visits completed, (SD) [range]	5.2 (1) [4–6]	5 (0) [5–5]	4 (0.6) [3–5]	0.0291 *
Visit 1 MMSE, mean (SD)	27.3 (1.9)	26.8 (3.3)	22 (1.8)	0.1609

Abbreviations: AD, Alzheimer’s disease; CDR, clinical dementia rating; MCI, mild cognitive impairment; MMSE, Mini mental state exam; NA, not applicable; SD, standard deviation. * *p* < 0.05, *** *p* < 0.001.

**Table 2:** MCI patient stratification and correlation of plasma-based candidate miRNAs according to cognitive decline and dementia.

**Age Adjusted FCSRT MCI Cut-Off Groups**	**MCI Above Cut-Off (*n* = 12)**	**MCI Below Cut-Off (*n* = 5)**	***p* Value**
Free Cued Selective Recall Test, mean (SD)			
Free Score at first sampling	14.3 (5.6)	8.4 (3.2)	0.0445 *
Free score annual change	1.4 (5.0)	−0.1 (5.2)	0.33
Free Score at final sampling	18.5 (7.1)	8 (3.2)	0.0059 **
Total score at first sampling	29.5 (6.9)	24.4 (6.6)	0.186
Total score annual change	2.2 (6.5)	−0.1 (6.8)	0.26
Total Score at final sampling	36.0 (6.2)	24.2 (7.2)	0.0037 **
Digit Symbol test, mean (SD)			
Score at first sampling	13.1 (5.8)	16.2 (4.8)	0.293
Annual change	−0.8 (2.9)	−1.2 (2.9)	0.39
Score at final sampling	10.73(6.0)	12.6 (4.7)	0.376
MicroRNA relative expression, mean (SD)			
miR-206	0.9 (0.7)	2.9 (1.9)	0.0174 *
Let-7b	2.1 (1.1)	2.5 (0.9)	0.68
miR-135a	1.2(1.3)	1.9 (1.6)	0.078
**MMSE Grouping**	**MCI Stable (*n* = 9)**	**MCI Decliners (*n* = 8)**	***p* Value**
Mini-Mental-State-Exam, mean (SD)			
Score at first sampling	27.6 (0.5)	26.0 (1.5)	0.0059 **
Annual change	−0.3 (2.4)	−1.3 (2.6)	0.16
Score at final sampling	26.6 (1.1)	22.0 (2.0)	< 0.0001 ***
Functional activities questionnaire, mean (SD)			
Score at first sampling	3.1 (1.4)	2 (1.4)	0.12
Annual change	−0.3 (2.0)	0.9 (2.8)	0.13
Score at final sampling	2.8 (1.8)	4.8 (6.0)	0.56
MicroRNA relative expression, mean (SD)			
miR-206	0.9 (1.0)	2.4 (1.5)	0.0055 **
Let-7b	2.4 (1.2)	3.1 (2.9)	0.72
miR-135a	1.2 (1.2)	1.6 (1.6)	0.9551

Abbreviations: FCSRT, Free Cued selective reminding test; MCI, mild cognitive impairment; MMSE, Mini mental state exam; NA, not applicable; SD, standard deviation. * *p* < 0.05, ** *p* < 0.01, *** *p* < 0.001.

## Supplementary Materials

The following are available online at https://www.mdpi.com/2218-273X/9/11/734/s1, Figure S1: MicroRNA profiling and absolute miR-206 concentrations in plasma, Figure S2: MCI analysis with Last observation carried forward, Figure S3: Development of a fast-electrochemical detection method for miR-206 in plasma.
