# Supplementary material for: Elevated Plasma microRNA-206 Levels Predict Cognitive Decline and Progression to Dementia from Mild Cognitive Impairment
Source: Biomolecules. 2019 Nov 13;9(11):734. doi: 10.3390/biom9110734 (PMC6920950; doi:10.3390/biom9110734)
Supplement: Supplementary file 1 [file biomolecules-09-00734-s001.pdf]

Supplementary Figure S1: MicroRNA profiling and absolute miR-206 concentrations in plasma

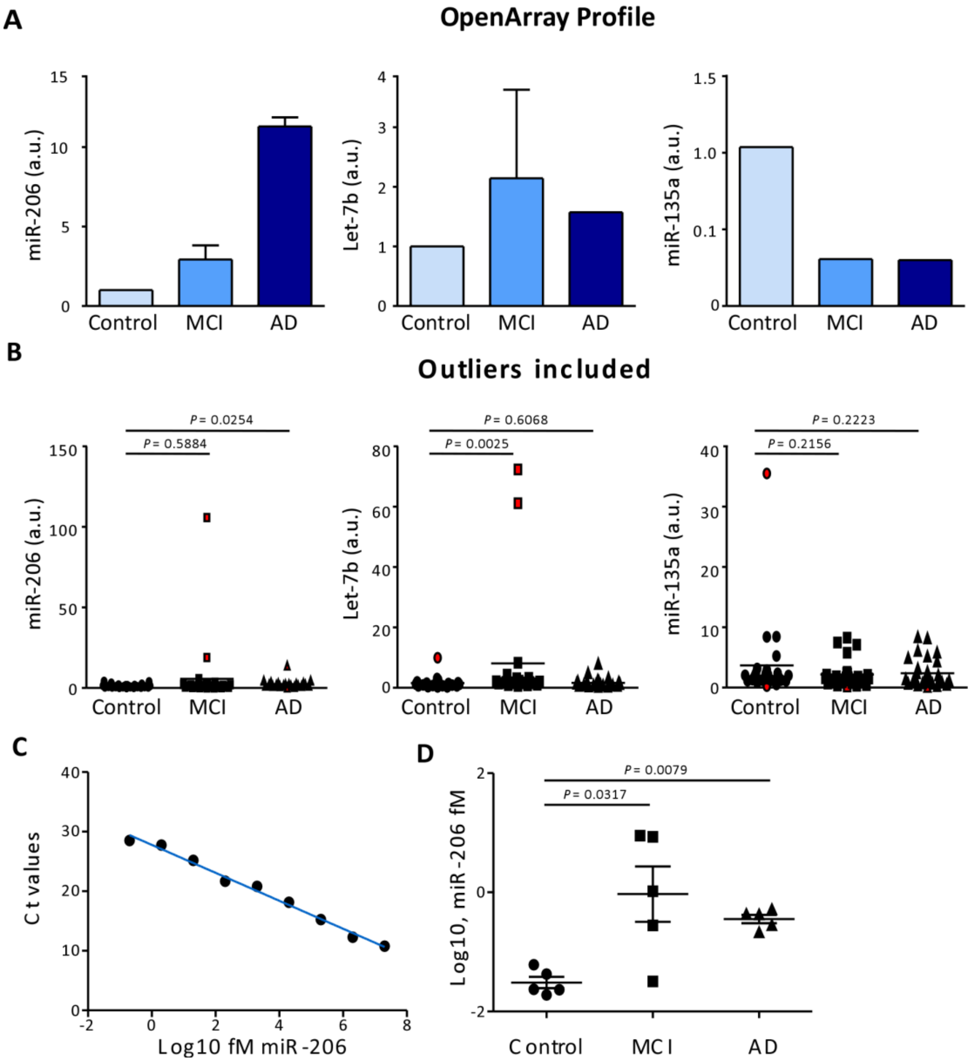

Figure S1. (**A**) Graphs showing increased miR-206 plasma concentrations in MCI and AD (left), increased Let-7b plasma concentrations in MCI (middle) and decreased miR-135a plasma concentrations in both MCI and AD (right) as detected by OpenArray (n = 2). (**B**) Individual RT-qPCR in validation cohort (with outliers included in red) shows increased miR-206 plasma levels in AD (left, MCI vs control *P* = 0.5884; AD vs control *P* = 0.0254; Mann-Whitney, n = 29 (control), n = 27 (MCI), 25 (AD)) and increased Let-7b plasma levels in MCI (middle, MCI vs control *P* = 0.0025; AD vs control, *P* = 0.6068, n = 24 (control), 22 (MCI), 23 (AD). No

differences were observed for miR-135a between conditions (right, MCI vs control $P = 0.2156$; AD vs control, $P = 0.2223$, n = 27 (control), 26 (MCI), 23 (AD)). (**C**) Graph showing standard curve of miR-206 using 9 serial dilutions (1:10) of miR-206 mimics ranging from 20 nM - 0.2 fM analysed by RT-qPCR and plotted as Ct value against Log10 of fentomolar concentration. (**D**) Concentration of miR-206 (fentomolar) in small RNA extracts from plasma in control, MCI and AD subjects (n = 5 per condition). RT-qPCR was run at same time as standard curve. MiR-206 is increased in plasma of MCI ($P = 0.0317$, Mann-Whitney, n = 5) and in AD ($P = 0.0079$, Mann-Whitney, n = 5). *$p < 0.05$, ** $p < 0.01$.

Supplementary Figure S2: MCI analysis with Last observation carried forward.

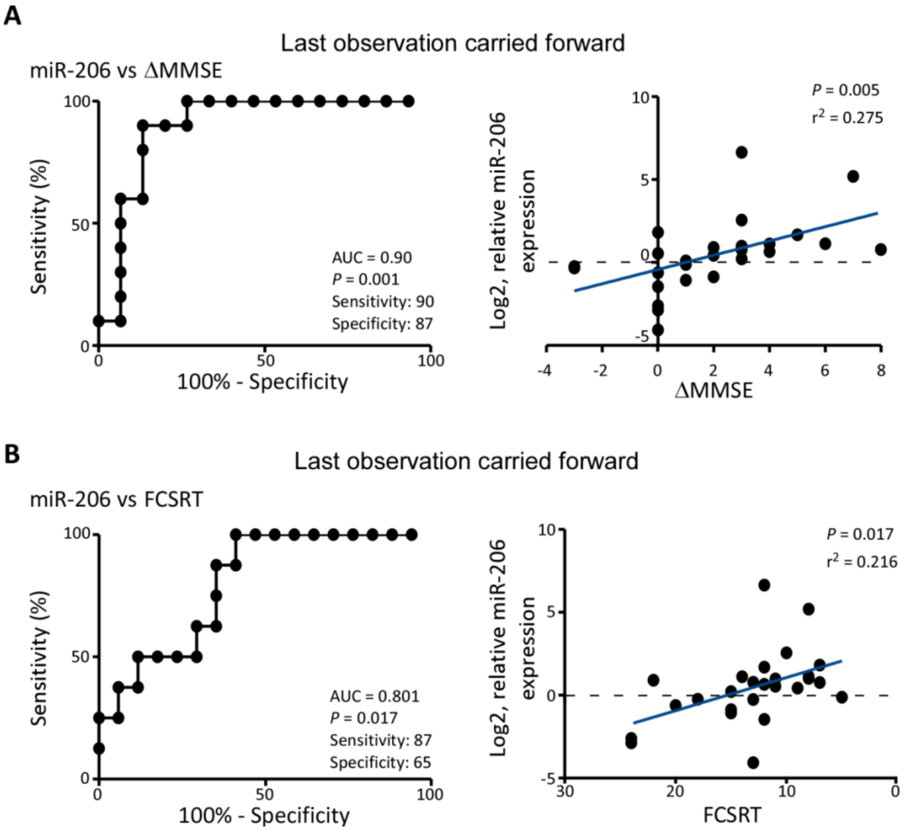

Figure S2. (**A**) MCI subjects with the inclusion of omitted subjects by Last observation carried forward (LOCF) method were grouped by changes in MMSE over 4 years. Relative miR-206 expression was significantly higher in cognitively declining group compared to stable MCI (*P* = 0.001; Mann-Whitney, n = 15 (MCI stable) and 10 (MCI decliners)) and plotting miR-206 agianst ΔMMSE showed a strong correlation (*P* = 0.005, n = 25). (**B**) MCI subjects including omitted subjects by LOCF method were grouped based on age-adjusted Free-cued-selective-reminding-test (FCSRT). Relative miR-206 expression was significantly higher in subjects below the FCSRT-free recall cut off (*P* = 0.017; Mann-Whitney, n = 17 (above cut-off) and 8 (below cut-off)) while relative expression of miR-206 showed a strong correlation with FCSRT-free scores (*P* = 0.017, n = 25).

Supplementary Figure S3: Development of a fast electrochemical detection method for miR-206 in plasma.

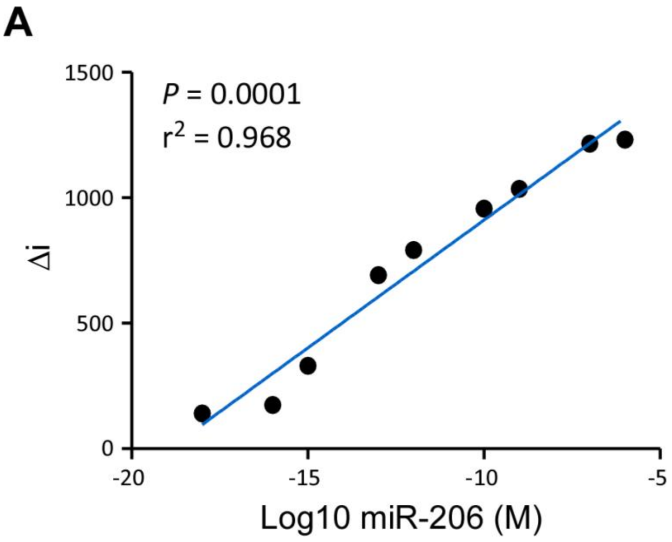

Figure S3: (A), Calibration curve for difference in current (Δi) before and after injection of hydrogen peroxide against known concentration of miR-206 oligonucleotides (linear between 100 nM – 100 aM).
